# Selective Nanoparticulate Systems for Drug Delivery in Inflammatory Bowel Disease

**DOI:** 10.3390/pharmaceutics18010055

**Published:** 2025-12-31

**Authors:** Alberta Ribeiro, Rute Nunes

**Affiliations:** 1 IUCS—University Institute of Health Sciences, Cooperativa de Ensino Superior Politécnico e Universitário (CESPU), Rua Central de Gandra, 1317, 4585-116 Gandra, Portugal; albertaleiterib@gmail.com; 2 UNIPRO—Oral Pathology and Rehabilitation Research Unit, University Institute of Health Sciences (IUCS), Cooperativa de Ensino Superior Politécnico e Universitário (CESPU), Rua Central de Gandra, 1317, 4585-116 Gandra, Portugal; 3 i3S—Instituto de Investigação e Inovação em Saúde, Universidade do Porto, Rua Alfredo Allen 208, 4200-135 Porto, Portugal

**Keywords:** cell targeting, inflammation, microenvironment-responsive nanoparticles, precision nanomedicine, targeted drug delivery systems

## Abstract

Inflammatory bowel disease is a result of inappropriate continuous non-specific inflammation in the intestinal tract, which in turn is aggravated by defects in the activation of the mucosal immune system and in the barrier function of the intestinal epithelium. The most prominent manifestations of IBD are ulcerative colitis (UC) and Crohn’s disease (CD). UC is characterized by a continuous pattern that commonly starts with lesions in rectum mucosa and is contained in the colon. On the other hand, CD affects the ileum and colon in a discontinuous pattern, and the lesions are often transmural. Conventional therapies often face limitations such as systemic side effects, poor drug stability, and low site-specificity. In recent years, nanoparticle (NP) systems have emerged as a promising strategy to overcome these challenges, offering improved targeting, controlled release, and enhanced therapeutic efficacy. Several studies have shown that the preferential accumulation of NPs in the inflamed colon is influenced by the pathophysiological changes associated with IBD, including alterations in transit time, pH value, enzymatic activity, microbial composition, and mucus integrity. These disease-specific characteristics provide unique opportunities to design smart and responsive NPs that enhance drug delivery and therapeutic efficacy while minimizing systemic exposure. This work presents an overview of novel technologies based on nanosystems, with the ability to specifically target the affected areas of the GI tract in inflammatory bowel disease.

## 1. Introduction

Crohn’s disease (CD) and ulcerative colitis (UC), the two main forms of inflammatory bowel disease (IBD), are chronic relapsing inflammations of the gut. In recent decades, the prevalence of IBD has been substantially increased throughout the world, affecting not only people in high-income countries but also in newly industrialized countries. From 1990 to 2017, the prevalence of IBD increased 85% worldwide, evolving into a global disease [1]. Owing to the young age of onset, low mortality, and lack of cure, it is estimated that the number of people with IBD will rise exponentially, representing a substantial burden on public healthcare [2]. The direct costs of managing the 2.5–3.0 million people suffering from IBD in Europe are estimated to be around EUR 5–6 billion annually [3]. More importantly, more than 20% of patients suffering from IBD will develop colorectal cancer within 30 years of disease onset [4].

The pathogenesis of IBD is poorly understood, but genetic and environmental factors seem to contribute to the loss of intestinal immune homeostasis, leading to chronic inflammation [5]. Currently, there is no cure for IBD, and the treatment relies mainly on anti-inflammatory and immunosuppressive agents with local or systemic action. Conventional treatment of acute episodes of disease consists of the daily administration of high doses of 5-amynosalicylates (5-ASAs) or corticosteroids drugs. However, conventional dosage forms fail to provide enhanced and sustained drug levels at the site of inflammation, leading to inconsistent efficacy, frequent dosing schedules, and severe side effects. In recent years, biologics (especially anti-tumor necrosis factor (TNF)-α agents) have revolutionized the treatment of moderate-to-severe IBD not responsive to conventional therapy or with extensive small bowel involvement in CD. However, the need for parenteral administration and serious side effects compromises their efficacy [6].

Drug-targeting technologies are urgently needed to deliver drugs directly to the affected area of the gastrointestinal (GI) tract or even cells of interest for improved efficiency and desired biodistribution profiles. Suitable engineering of nanosystems allows the selective delivery of drugs to the sites and/or cells of interest, either by receptor–ligand, physical, or chemical targeting [7]. This manuscript provides an up-to-date and concise overview of the development of targeted nanotechnology-based systems for inflammatory bowel diseases treatment.

## 2. Etiology and Pathophysiological Mechanisms of Inflammatory Bowel Disease

**Figure 1:**
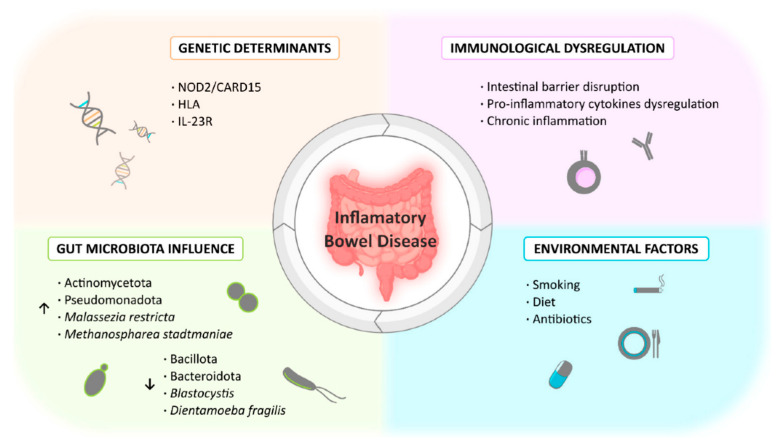
Factors contributing to inflammatory bowel disease. Reproduced from reference [27].

The precise mechanism behind the onset of IBD is not fully understood. Rather than being caused by a single factor, IBD seems to result from a complex interplay between genetic, microbial, epithelium-mediated, immunological, and environmental factors (Figure 1).

Familial aggregation and twin studies have established a genetic contribution to the development of IBD, but the heritable component seems to be stronger in CD than in UC [8]. Specific gene variations that increase susceptibility to IBD have been identified [9,10]. The first gene to be associated with IBD was NOD2 (nucleotide-binding oligomerization domain-containing protein 2), encoding a protein that recognizes molecular patterns present in bacteria and activates signaling pathways that modulate immune responses [11]. Mutations in the NOD2 gene result in unresponsiveness to pathogens, allowing them to penetrate the gut and trigger an immune inflammatory response [12]. Other genes associated with the immune response, detection of pathogens, autophagy (ATG16L1, IRGM), phagocytosis, or intestinal barrier integrity (MUC19) have been identified as having a role in the development of IBD. Disruption in the function of many of these genes increases the risk of development of IBD, but also of other inflammatory disorders [13]. Although genetic factors are important, no single genetic variant can explain the development of IBD on its own. The fact that most individuals who carry IBD-associated risk variants remain healthy, while others develop IBD, emphasizes this inference. Clearly, genetics alone is unable to explain the susceptibility and progression of IBD.

Nonetheless, several environmental factors have been linked to an increased risk of IBD in genetically susceptible individuals. These include smoking, diet, and the use of certain medications [14,15,16]. The effects of smoking have been extensively studied and described as a risk factor for CD. In contrast, smoke seems to exert a protective role in UC [17]. Exposure to diets rich in saturated fatty acids and processed foods has been reported to increase the risk of IBD. Conversely, a high-fiber diet has been found to reduce the risk of CD [18,19]. The use of certain medications, such as non-steroidal anti-inflammatory drugs, antibiotics, and contraceptives, is also associated with an increased risk IBD [20]. Direct toxicity to immune- and mucus-producing cells, autophagy impairment, and induction of microbiome dysbiosis are some of the mechanisms by which environmental factors contribute to IBD [21].

Microbiome dysbiosis is one of the most important mechanisms triggered by environmental factors. Dysbiosis is defined as an imbalance of gut microbiota, with consequences in its composition and function. This imbalance is characterized by (a) a decrease in beneficial microbiota species, (b) an increase in pathogenic microorganisms, (c) lack of diversity in microbiota species, or, more frequently, (d) the combination of all these features. Microorganisms of a healthy microbiota have a crucial role in the normal immune response, modulating it and maintaining its homeostasis along with the intestinal epithelial barrier [22]. Microbiota dysbiosis has been observed in IBD patients. IBD patients present a decrease in bacteria that produce short-chain fatty acids (SCFAs), such as butyrate, propionic acid, and acetate, which have anti-inflammatory effects. Conversely, it can be observed that there is an increase in bacteria with pro-inflammatory properties [22,23]. Regardless of whether it is a cause or consequence of the disease, microbiota appears to play a major role in the development and progression of the disease.

Gut microbiota in patients with IBD have less biodiversity when compared to normal patients [24]. These cases have an increased quantity of Escherichia coli, which is a pathogenic bacteria, and an increase in mucolytic bacteria, like *Ruminococcus gnavus*. There are also some bacteria that can decrease, such as *Firmicutes* and *Clostridium*, which play a big role in the diversity and regulation of the microbiota [25,26].

The intestinal epithelium is composed of a protective mucus layer enriched with antimicrobial molecules, a layer of intestinal epithelial cells (IECs), and a set of supporting cells [28]. This structure plays important role in mediating the crosstalk between the intestinal microbiota and the host immune system, largely through bidirectional signaling between microbial communities and IECs. In IBD, this communication is disrupted by increased epithelial permeability, which represents a key mechanism contributing to disease pathogenesis [29].

The inflammatory response in IBD is different when comparing ulcerative colitis and Crohn’s disease. In UC, CD4+ lymphocytes possess a phenotype for a type 2 helper T-cell (Th2) that is responsible for the production of transforming growth factor β (TGF- β) and interleukin-5. Alternately, CD is characterized by CD4+ lymphocytes with the phenotype for a type 1 helper T-cell (Th1) that is responsible for the production interferon-γ and interleukin-2 [5,13,26].

Cytokine pathways in IBD can be divided by innate immunity and adaptative immunity. Innate immunity is characterized by the release of TNF-related cytokines, Il-6, and Il-1 [30]. TNF is a cytokine that is capable of inducing apoptosis in tumor cells and exerting a wide range of pro-inflammatory actions [31]. These responses are mediated by the TNFR1 and TNFR2 receptors [30]. This process results in immune system dysregulation driven by T-cells, characterized by enhanced effector T-cell activity and the subsequent recruitment of additional immune cells that amplify the inflammatory response [32]. Within the TNF superfamily, another important member is TL1-A (TNF ligand-related molecule 1A), which binds to the death receptor 3 (DR3), a receptor capable of activating Map kinases [33]. In IBD, both TL1-A and DR3 are upregulated, and due to the widespread expression of TL1-A across multiple cell types, it is challenging to attribute its specific endothelial expression [34].

IL-6 increases intestinal permeability by targeting the tight junction protein Claudin-2, which facilitates the passage of small cations across the epithelial layer [35].

On the other hand, adaptive immunity is characterized by the involvement of IL-12, IL-23, and Th17 cells. IL-12 and IL-23 share the p40 subunit, with IL-23 also containing the p19 subunit. IL-12 primarily acts on naïve T-cells, while IL-23 plays a role in memory T-cells [36,37]. Th17 cells have an active participation in the development of inflammation in IBD. CD4+ lymphocytes can differentiate in Th17 cells by stimulation of IL-6 and TGF-β, and subsequently, Th-17 cells secrete IL-17, IL-21, IL-22, and TNF-α. IL-17 and IL-21 in patients with IBD can stimulate the secretion of proteases that in return will cause epithelial cell damage. IL-21 can also promote more differentiation of Th17-cells and more secretion of IL-17 [38]. Il-23 is also associated with the stimulation, and proliferation of Th-17 cells [13].

## 3. The Gastrointestinal Environment in Inflammatory Bowel Disease

### 3.1. pH

Under normal conditions, the gastrointestinal tract exhibits pH variations depending on the specific region. Typically, the stomach has an acidic environment (pH 1 to 3). In the small intestine, the environment becomes less acidic and more neutral (pH 6.5 to 7). The proximal colon is acidic again (pH 5 to 7), and finally pH rises in the rectum (pH 7–8) [39]. In IBD, pH levels can change. In the small intestine, pH remains relatively unchanged; however, in the colon, a decrease in pH (pH values around 2.3–5.5) has been observed compared to a healthy individual [40].

### 3.2. Mucus

**Figure 2:**
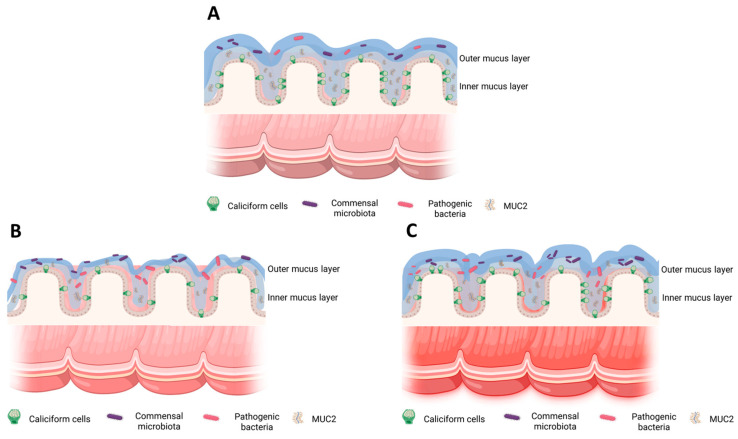
Intestinal mucosa in health and disease conditions. (**A**) Healthy intestinal mucosa. (**B**) Intestinal mucosa in ulcerative colitis and (**C**) intestinal mucosa in Crohn’s disease. Created with Biorender.com.

Mucus is a layer that surrounds the intestinal epithelium and serves to protect the epithelium from damage, preventing the binding of pathogens to the epithelial surface, and provide lubrication (Figure 2). These functions are made possible by goblet cells, which are responsible for secreting mucus [41]. The colonic mucus comprises two distinct layers: a basal layer, which is more dense and adherent to the epithelial surface and resistant to bacteria, and an outer, looser layer located in the luminal space [42,43]. The inner, firmly attached mucus layer is continuously renewed and feeds the non-adherent outer mucus layer, which harbors a vast community of intestinal commensal bacteria [44]. In IBD, due to the loss of epithelial integrity and tissue ischemia, protective pathways are activated as a compensatory response. However, this activation can paradoxically lead to a reduction in mucus production [39,42]. Typically, UC is characterized by a decrease in goblet cells number, a reduction in MUC 2 expression, and a thinning mucus layer [45]. In contrast, goblets cells hypertrophy is seen in CD with an increased mucus production, which can be up to three times more compared to healthy individuals [46].

### 3.3. Enzymes

Compared to healthy individuals, the enzyme profile in the GI tract and small intestine exhibits substantial alterations in patients with IBD. These changes are primarily characterized by increased activity of certain enzymes, including proteases and lipases. Enzymes derived from intestinal microbiota also undergo significant modulation and may represent critical targets for therapeutic intervention in IBD [47]. Enhanced proteolytic activity contributes to disease progression and is positively correlated with IBD severity and activity. Elevated levels of tryptase, for instance, can activate pro-inflammatory cytokines, and promote intestinal fibrosis, thereby exacerbating disease activity [48].

Matrix metalloproteinases (MMPs) are zinc-dependent endopeptidases that cleave components of extracellular cellular matrix, promoting cellular turnover. MMPs depend on TIMPs, their inhibitors, to function [49]. In IBD, the expression of MMPs is increased and TIMP-1 is reduced, which indicates an inability to suppress MMPs activities [50].

### 3.4. Intestinal Epithelium

The intestinal epithelium acts as the physical barrier against harm. This layer is composed of specialized IECs, such as goblet cells, Paneth cells, and many others. So, in a healthy individual, this maintains balance between microbiota and immune cells [51]. In patients with IBD, the inflammatory response often results in continuous epithelial injury, leading to ulcers and erosions that compromise the intestinal barrier. Intestinal barrier dysfunction is a hallmark of IBD, though the exact relationship between the barrier defects and chronic inflammation is complex, with defects potentially acting as both a cause and a consequence [52,53]. At the same time, defects in IECs can play a significant role in the development and progression of IBD.

Beyond their absorptive function, enterocytes are actively involved in shaping the intestinal immune environment by secreting cytokines and interacting with immune cells [54]. Goblet cells are mucus-secreting cells, assuring the presence of a continuous mucus layer that protects the intestine against harmful substances or microorganisms. Furthermore, goblet cells play an important role in immune regulation. Paneth cells contribute to epithelial repair and regulation of inflammation, by secreting chemokines, cytokines, and antimicrobial peptides, such as α-defensins [55]. Reduced secretion of α-defensins by Paneth cells was found in patients with ileal CD [56,57].

Barrier dysfunction is driven by altered epithelial permeability, particularly its increase under inflammatory conditions [52]. Epithelial permeability is regulated both at cellular level and through tight junctions (TJs). An intact intestinal epithelium depends on tight junctions, which help seal the space between adjacent epithelial cells (the paracellular space). TJs are composed of claudins, proteins that form cation-selective channels. These channels can respond to immune-mediated signals, resulting in increased permeability. Such alterations facilitate translocation of luminal antigens, thereby perpetuating disease progression [58]. Increased intestinal permeability has been reported in IBD patients [59,60,61,62]. The impairment of intestinal permeability is assigned to different molecular mechanisms, including altered expression and structural changes in tight junctions [63,64].

### 3.5. Intestinal Transit Time/Mobility

In healthy individuals, the median gastric transit time is approximately 22 min, although this value can be influenced by factors such as diet, fasted or fed state, and usual physical activities [65]. In UC, an increase in transit time is often observed. Regarding intestinal contractions, despite the prolonged transit time, bowel movement frequency may also be increased. This apparent paradox can be explained by the fact that not all contractions are propulsive; therefore, the higher number of contractions does not necessarily result in faster transit. An explanation for the increased transit time, despite more frequent bowel movements, is that the latter may reflect episodes of frequent and urgent defecation rather than efficient propulsion. Rectal distension can trigger reflex pathways that slow transit in the proximal segments of the gastrointestinal tract, thereby contributing to the overall delay [66].

### 3.6. Intestinal Volume

In IBD, chronic inflammation impairs water and electrolyte absorption, thereby disrupting the osmotic gradient and promoting luminal water retention, which results in diarrhea. Electrolyte imbalance and altered intestinal volume are attributed to dysregulation of sodium transport. Sodium hydrogen exchangers (NHEs) mediate the exchange of Na^+^ for H^+^. In IBD, NHE3 activity is significantly reduced, rendering patients more susceptible to diarrhea. The sodium potassium ATPase also contributes to the development of disturbances in intestinal volume and further exacerbates sodium malabsorption.

In addition to sodium, chloride transport is also impaired. The principal intervenient is Downregulated in Adenoma (DRA), which is the main transporter mediating CL^−^/HCO_3_^−^ exchange. Reduced DRA expression and function have been documented in IBD and lead to chloride malabsorption and diarrhea [67].

### 3.7. ROS Levels

Reactive Oxygen Species (ROS) are a well-recognized hallmark of inflammatory disorders. They are generated through the reduction of molecular oxygen to superoxide dismutase. Excessive accumulation of these species decreases activity of nitric oxide, thereby promoting endothelial cell dysfunction. Oxidative stress plays a pivotal role in the pathogenesis and progression of IBD, and elevated concentrations of ROS have been reported in diseased tissues of IBD patients [68,69,70,71,72].

In active disease, infiltration of innate immune cells further amplifies ROS production. These cells release nitric oxide and superoxide, which combine to form peroxynitrite, a potent oxidant that triggers apoptosis. Persistent ROS overproduction contributes to cellular damage and enhances inflammatory responses [73].

## 4. Limitations of Current Dosage Forms for the Delivery of Inflammatory Bowel Disease Drugs

Conventional treatment of IBD typically involves aminosalicylates, corticosteroids, immunomodulators, and biologics, along with additional measures or surgical intervention [74]. The specific approach is based on disease severity and extent, location, and previous treatment history. Treatment goals are focused on controlling inflammation, avoiding complications, and limiting disease progression [75,76]. Two steps can be followed: the “step-up” and the “top-down”. In the first case, disease flares will first be treated with aminosalicylates or corticosteroids. In case of failure, or to achieve steroid-free remission, immunomodulators, biologicals, and small-molecule inhibitors will be considered. In the “top-down” approach, the treatment starts with immunomodulators, biologics, or a combination of both. The “step-up” approach is recommended in the recent guidelines of the European Crohn’s and Colitis Organization (ECCO), with evidence suggesting top-down may be more effective for achieving and maintaining remission, especially in certain cases with poor prognostic outcome, by preventing long-term complication [74,77].

Therapeutic agents can be delivered through oral, parenteral, or rectal routes, each with distinct advantages and limitations (Table 1).

Oral administration remains the most common, due to its convenience for self-administration, favorable safety profiles, and flexibility in dose adjustment. However, oral formulations are more highly susceptible to degradation by gastric acid and gastrointestinal enzymes prior to total absorption, which may compromise the therapy [68]. To overcome this limitation, aminosalycilates and glucorticoids (ex. budesonide, methylprednisolone) are orally administered to IBD patients using strategies aiming to targeting the colon including colonic-bacteria-dependent prodrugs (ex. Azulfidine^®^, Sulfasalazine) or pH-dependent drug delivery systems (ex. Salofalk^®^, Mesalazine; Entocort^®^, budenoside) [78].

Injectable administration, particularly subcutaneous and intravenous injections, ensures high bioavailability and a reduced first-pass metabolism when compared to oral administration [68,79]. Nevertheless, at-home administrations of injectable therapies may induce fear and discomfort, thereby negatively affecting patient adherence [80]. Repeated injections can result in accumulation and an increased risk of systemic adverse effects.

Rectal administration, although underutilized, provides targeted delivery to the inflamed mucosa, offering improved local efficacy with reduced systemic toxicity. This route is particularly relevant for distal colonic disease, where localized drug deposition enhances therapeutic outcomes [68]. Rectal enemas, foams, gels, and suppositories are commonly used to local deliver 5-ASA and corticosteroids to treat IBD [81,82,83]. However, rectal dosage forms provide short retention time in diseased sites due to rectal leakage, requiring frequent administration. This may be accompanied by local irritation, discomfort, or increased defecation urges, thus rendering them unacceptable by some patients [81,84].

The lack of specificity, low bioavailability, incomplete response rates, loss of efficacy over time leading to recurrence, and significant side effects of current IBD medications imposes continuous research for the development of novel drug delivery systems addressing these gap [85].

**Table 1:** Pharmacological therapy in inflammatory bowel disease [68,79,80,81,82,83,84,85].

Class	Drug		Disease	Route of Administration	Common Adverse Effects
Aminosalicylates	Sulfasalazine		UC	Oral, Rectal	Headache, dizziness, dyspepsia, epigastric pain, abdominal pain, nausea, vomiting, and diarrhea.
	Balsalazide		Mild-to-moderated UC		
	Mesalamine		Mild-to-moderated UC		
	Olsalazine		UC		
Glucocorticoids	Prednisolone		Moderated-to-severe CD, UC	Oral	Full moon face, difficulty of healing, acne, mood and sleep disturbances, glucose intolerance, osteoporosis, osteonecrosis, subcapsular cataracts, myopathy, infections, acute adrenal insufficiency, myalgia, malaise, arthralgia or intracranial hypertension, and pseudorheumatism.
	Prednisone		Moderated-to-severe CD, UC	Oral	
	Methylprednisolone		Moderated-to-severe CD, UC	Oral, IV	
	Budesonide		Mild-to-moderate CD, UC	Oral, Rectal	
Immunomodulators	Azathioprin		CD, UC	Oral	Black, tarry stools, bleeding gums, chest pain, fever, chills, swollen glands, pain, cough, and weakness.
	6-mercaptopurin		CD, UC	Oral	
	Cyclosporin		UC	Oral, IV	
	Tracolimus		Active CD	Oral, IV	
	Methotrexate		Moderate-to-severe CD, UC	Oral, SC	
Biologics	Anti-TNFα	Infliximab	Moderate-to-severe CD, UC	SC, IV	Abdominal or stomach pain, chest pain, chills, cough, dizziness, fainting, headache, itching, muscle pain, nasal congestion, nausea, sneezing, weakness, vomiting, bloody urine, cracks in the skin, diarrhea, pain, fever, abscess, back or side pain, bone or joint pain, constipation, falls, facial edema, general feeling of illness, hernia, irregular heartbeat, unusual bleeding, weight loss, increased risk of reactivation of latent tuberculosis, increased risk for developing infections, and lymphoma.
		Adalimumab	CD, UC	SC	
		Golimumab	UC	UC	
		Certolizumab pegol	Moderate-to-severe CD	SC	
	IL-12/23 inhibitors	Ustekinumab	CD, UC	IV	
		Risankizumab	CD	SC	
		Mirikizumab	Moderate-to-severe CD	IV	
	CAM inhibitors	Natalizumab	Moderate-to-severe CD	IV	Nasopharyngitis, headache, abdominal pain, increased risk of serious infections, and progressive multifocal leukoencephalopathy in Natalizumab.
		Vedolizumab	CD, UC	SC, IV	
Small Molecules	JAK inhibitors	Tofacitinib	UC	Oral	Increased risk of infection, hyperlipidemia, venous thromboembolism, and cytopenias.
		Upadacitinib	Moderate-to-severe CD		
	S1P modulators	Ozanimod	Moderate-to-severe CD	Oral	Infections, leukopenia, and bradycardia.
		Etrasimod	UC		

Abbreviations: CAM: cell adhesion molecule; CD: Crohn’s disease; IL: interleukin; IV: intravenous; JAK: Janus kinase; S1P: sphingosine 1-phosphate; SC: subcutaneous; TNF-α: tumor necrosis factor alpha UC: ulcerative colitis.

## 5. Nanoparticulate Drug Delivery Systems for IBD Treatment

Novel drug delivery strategies increasingly rely on drug-loaded nanoparticles, which enable targeted delivery to specific tissues while sparing healthy cells, thereby minimizing systemic toxicity and enhancing therapeutic efficacy. These nanocarriers can be engineered to respond according to disease stage and pathological situation, allowing for optimized dosing schedules with lower doses and reduced administration frequency. Such features promote accumulation at the site of inflammation, further improving treatment outcomes and overall efficiency [86].

Drug delivery platforms explored in IBD include polymeric NPs, lipid-based NPs, metallic NPs, and exosomes (Table 2).

Polymeric NPs are particularly versatile, as their physiochemical properties, such as their small sizes, shape, and surface charge, can be finely tuned to optimize drug delivery. Moreover, they are capable of encapsulating a broad range of therapeutic molecules expanding their applicability in IBD therapy [87].

Lipid-based nanoparticles (LNPs) represent a diverse class of carriers that include Solid Lipid Nanoparticles (SLNs), Nano-Structured Lipid Carriers (NLCs), Liposomes, and Polymer–Lipid Hybrid Nanoparticles (PLNs). SLNs are characterized by a solid lipid core stabilized by a surfactant, enabling the encapsulation of both hydrophilic and lipophilic compounds. Their dimensions provide a high surface area-to-volume ratio, thereby enhancing solubility and, ultimately, drug bioavailability [88].

Metallic nanoparticles (MNPs), commonly composed of silver or gold, exhibit unique physicochemical characteristics that expand their potential in IBD therapy. Their optical properties make them valuable tools for both diagnostic imaging and targeted drug delivery. In addition, MNPs display intrinsic antibacterial and anti-inflammatory effects. Owning to their nanoscale size, these particles can efficiently traverse biological, enabling precise delivery to tissues [89].

Exosomes are nanoscale membranous vesicles enclosed by a lipid bilayer and secreted by a wide range of cell types. They are critical mediators of intercellular communication and play an key role in modulating immune responses [90]. Owing to their biocompatibility and endogenous origin, exosomes have emerged as promising drug delivery vehicles in IBD. Notably, they enhance the solubility and anti-inflammatory activity of therapeutic agents while exhibiting low immunogenicity, which further supports their potential for clinical application [91].

**Table 2:** Advantages and challenges of different classes of nanoparticles and examples of their applications in the treatment of inflammatory bowel disease [92].

Nanoparticulate DDS	Advantages	Challenges	Examples of Its Application in IBD
Polymeric nanoparticles	Versatility Biodegradability and biocompatibility (depending on the polymers used) Protection of cargo against degradation Possibility to modify the release profile Easy functionalization	Limited loading efficiency (especially for large biomacromolecules) Potential toxicity (non-biodegradable polymers) Unpredictable clearance.	Polymeric nanoparticles based on the synthetic methacrylate-based copolymer Eudragit S100 and coated with HA (size~275 nm, ZP~−25 mV and AE~98%) improved BUD therapeutic efficacy and IBD symptoms in a rat model of induced colitis [93]. Mucus-penetrating PEG-PLGA NPs encapsulating cyclosporine A provided higher concentrations of the drug in intestinal inflamed tissues of a rat model of chemical-induced colitis, as well as exhibited anti-inflammatory effects after repeated oral administrations [94].
Lipid-based nanoparticles	Biocompatibility and biodegradability Versatility Low toxicity Simple manufacturing Possibility to modify the release profile	Low encapsulation efficiency for some molecules Fast clearance (need surface PEGylation) Susceptible to physiological conditions	TNF-α siRNA was entrapped (AE~97%) in LNPs composed of Dlin-MC3-DMA, DSPC, cholesterol, and PEG2000-C-DMG (size~150 nm, ZP~−1.4 mV). siRNA-loaded LNPs were further microencapsulated within alginate microparticles and orally administered to mice with TNBS-induced colitis. LNPs in MPs were able to target the inflamed colon, significantly reducing the clinical score (as assessed by survival and weight loss) and providing a therapeutic index similar to that observed in healthy animals [95]. LNPs produced with newly developed ionizable lipids, cholesterol, DSPC, and PEG-DMG (size 50–70 nm, ZP between −6 and 10 mV) were used to deliver ASOs, namely, LNAs to the inflamed colon of mice after retro-orbitally injection. ASO-LNA-loaded LNPs showed 10-fold-enhanced accumulation in the inflamed colons compared to healthy tissue with a consequent decrease in inflammatory cytokines and clinical symptoms (assessed by weight loss and colon length) of DSS-induced colitis mice [96].
Metallic nanoparticles	High stability Unique properties (ex. optical properties) Suitable for simultaneous diagnostic and therapeutic applications	Potential toxicity Limited biodegradability Hard clearance with potential accumulation in tissues	In vivo CT imaging showed that Dextran-coated cerium oxide nanoparticles provided better contrast in the GIT of mice with DSS-induced colitis compared to iopamidol. Additionally, Dex-CeNPs administered via oral gavage significantly decreased DAI scores and diminished GIT bleeding in the same model when compared with 5-ASA [97].
Exosomes	High stability Low immunogenicity Biocompatibility Reduced toxicity Enhanced bioavailability [98]	Batch-to-batch invariability Lack of standardization for isolation, purification and storage Low yield for large scale manufacturing Regulatory issues [99]	miR-146a-loaded EVs significantly suppress inflammation by downregulating TRAF6 and IRAK1 in TNBS-induced colitis mice model [100].

Abbreviations: 5-ASA: 5-aminosalicylates; AE: association efficiency; ASOs: antisense oligonucleotides; BUD: budesonide; CT: computed tomography; DAI: disease activity index; DDS: drug delivery systems; Dex-CeNPs: Dextran-cerium oxide nanoparticles; DMG: 1,2-dimyristoyl-sn-glycerol; DSPC: distearoylphosphatidylcholine; DSS: dextran sodium sulfate; EVs: extracellular vesicles; GIT: gastrointestinal tract; HA: hyaluronic acid; IBD: inflammatory bowel disease; IRAK1: IL-1 receptor-associated kinase 1; LNAs: locked nucleic acids; LNPs: lipid nanoparticles; miR: microRNA; MPs: microparticles; PEG: polyethylene glycol; PLGA: poly(lactic-co-glycolic acid); siRNA: small interfering RNA; TNF-α: tumor necrosis factor alpha; TRAF6: TNF receptor-associated factor 6; TNBS: 2,4,6-trinitrobenzenesulfonic acid; TNF: tumor necrose factor; ZP: zeta potential.

### 5.1. Nanoparticulate Drug Delivery Systems for Selective Targeting of Intestinal Inflammation

#### 5.1.1. Size-Mediated Targeting

Nanoparticle size is a critical determinant for effective targeting of inflamed tissues. Owning to the enhanced epithelial permeability and retention (eEPR) effect observed in inflamed sites, smaller nanoparticles (100–200 nm) are able to passively transverse epithelial membranes, thereby facilitating their accumulation at the site of inflammation [100]. Lamprecht et al. [101] initially demonstrated that 100 nm polystyrene particles adhere more effectively to TNBS-induced inflamed colonic tissue compared to larger counterparts (1 µm, 10 µm) [78]. Complementing these findings, a redox- and pH-responsive lipid-like nanoparticle system showed size-dependent accumulation in DSS-induced colitis; mid-sized NPs (~113 nm) accumulated most efficiently in inflamed regions [102]. Defects in the epithelial barrier, altered mucus production, and marked infiltration of immune and inflammatory cells explain the preferential accumulation of NPs in inflamed tissues [101].

Small to mid-sized nanoparticles (<200 nm) demonstrate superior ability to penetrate the mucus layer, which is typically compromised under inflammatory conditions in IBD [102]. In contrast, larger nanoparticles (500–1000 nm) exhibit reduced transepithelial transport but increased retention within Payer’s patches, suggesting potential applications for localized immune modulation rather than deep mucosal penetration [103]. Importantly, reviews highlight that size interacts with other physicochemical properties to modulate nanoparticle and cell interactions, influencing cellular uptake, intracellular trafficking, and immune response within the intestinal mucosa [104].

Overall, these observations highlight that nanoparticle size is a complex and context dependent factor in IBD targeting. Rather than defining a single “ideal” size, it suggests that different sizes may be strategically employed for distinct therapeutic purposes, and that further exploration is needed to fully exploit size-dependent behaviors in inflamed tissues.

#### 5.1.2. Charge-Mediated Targeting

Surface charge of NPs plays a critical role in determining their therapeutic efficacy in IBD by influencing interactions with the intestinal mucus, epithelial penetration, and modulation of the immune response.

Cationic NPs, due to their positive surface charge, exhibit enhanced adhesion to the negatively charged mucins (due to sulfate and sialic acid groups) in the intestinal mucus, leading to increased local retention and sustained drug release [105]. For instance, positively charged polymethacrylate (Eudragit RL^®^)-based NPs (~120 nm, +26 mV), loaded with clodronate (~93%), demonstrated reduced myeloperoxidase activity in murine colitis models, attributed to electrostatic interactions with the mucosal surface [106,107].

In turn, anionic NPs have garnered considerable interest in the context of IBD due to their favorable interactions with the inflamed intestinal mucosa. Studies performed with negatively charged liposomes (800 nm; −66 mV) have demonstrated preferential adhesion to DNBS-induced inflamed colonic tissues in rats, compared to healthy colon tissues. Compared to their cationic or neutral counterparts, anionic liposomes demonstrated higher adhesion to inflamed tissues [108]. Furthermore, anionic liposomes enhanced the activity of antioxidant molecules (catalase, superoxide dismutase (SOD), and SOD-mimic tempamine) following intracolonic administration to rats with DNBS-induced colitis [109]. The infiltration of immune cells into the mucosa leads to increased concentration of positively charged proteins (e.g., eosinophil cationic proteins, transferrin, and antimicrobial peptides) in inflamed sites, which, combined with a reduction in negative charges due to mucus deterioration (in the case of UC), explains the preferential target of anionic systems to injured tissues [110].

While cationic NPs increase the adherence within the mucus layer and anionic NPs increase the interaction with the injured epithelial barrier, neutral NPs have proved to be important to circumvent the mucus barrier due to the lack of interactions with mucins. NPs with a near-neutral surface charge, often achieved through hydrophilic coatings like PEG, are crucial for crossing the intestinal mucus barrier once they minimize strong electrostatic interactions with the negatively charged mucin glycoproteins, allowing them to diffuse through the mucus mesh instead of getting trapped or quickly cleared [111]. Several studies have demonstrated that a dense PEGylation of small NPs’ (50–200 nm) surfaces with a low-molecular-weight PEG (2–5 KDa) allows a rapid transposition of the intestinal mucus barrier [112]. Although initial studies were conducted on healthy tissues, mucus-penetrating NPs have also exhibited this property in inflamed intestinal tissues [113]. Using PEGylated mucus-penetrating NPs to deliver drugs such as budesonide [114,115], teduglutide [116], cyclosporine A [94], and anti-TNF-α antibodies [117] has been shown to enhance the resolution of colitis in vivo more effectively than standard treatments or free drugs. While a molecular weight of 5 kDa was generally recognized as optimal for achieving mucus-penetrating properties in PEGylated NPs, recent studies have shown that those with a molecular weight of 2 kDa (PLGA-PEG2k) were more efficient in alleviating inflammation in an acute murine colitis model than those with a molecular weight of 5 kDa (PLGA-PEG5k) [117]. Although PLGA-PEG2k and PLGA-PEG5k exhibited similar behavior regarding their interaction with mucins in vitro, the higher hydrophilicity of PLGA-PEG5k NPs decreased its interaction with inflamed intestinal tissue in mice. This resulted in lower efficacy of PLGA-PEG5k NPs in reducing inflammation when delivering an anti-TNF-α antibody, as indicated by a worse colon weight/length ratio, a lower histological score, and increased tissue-associated myeloperoxidase activity (MPO), compared to anti-TNF-α antibody-loaded PLGA-PEG2k NPs.

Cationic and anionic NPs can interact non-specifically with non-target tissues and cells, leading to unwanted adhesion that reduces therapeutic efficacy and increases the risk of toxicity. Cationic NPs, due to their positive surface charge, tend to adsorb plasma proteins and bind to endothelial cells, such as liver and spleen macrophages, resulting in accelerated clearance from circulation and reduced bioavailability at the inflamed target site [26,118].

These findings illustrate that NP surface charge is a nuanced determinant of therapeutic performance in IBD. While cationic and anionic NPs can be leveraged to enhance adhesion and targeting in inflamed tissues, they also carry the risk of off-target interactions and unwanted clearance. Further research is needed to explore how charge, in combination with other NP properties, can be fine-tuned to maximize efficacy and safety.

#### 5.1.3. Microenvironment-Responsive Nanoparticles

##### pH-Responsive NPs

pH-sensitive NPs have gained increased attention in IBD for their ability to selectively target colonic regions while avoiding premature degradation at non target sites. These nanosystems are designed to remain stable within acidic gastric environment, while releasing the therapeutic agents at colonic pH values above 7. NPs that enable colonic drug delivery are often produced using pH-dependent polymers like the polymethacrylate Eudragit^®^ S100 (Evonik, Essen, Germany) or the natural polymer chitosan (CS) [81].

For instance, Eudragit^®^ S100-coated mesoporous silica nanoparticles successfully protected glucocorticoids from premature release and achieved targeted drug delivery to the colon, reducing colitis severity in a DSS-induced colitis mice model [119]. Similarly, methotrexate-loaded PLGA NPs (~200 nm) coated with Eudragit^®^ S100 and chitosan provided colon-specific release after oral delivery, leading to enhanced local accumulation and macrophages target (due to HA surface modification), attenuating inflammation, and promoting mucosal repair in murine models [120]. The same NPs were used for the colonic delivery of a miR-301a inhibitor (anti-miR-301a (anti-miR)). The microRNA miR-301a plays an important role in the progression of IBD, and its inhibition has remarkable therapeutic effects. HA-CS/ES100/PLGA NPs were found to accumulate preferentially in the colon of IBD mice. The macrophage-targeting colonic delivery of anti-miR-301a promoted the repair of the intestinal barrier and controlled the intestinal inflammation of an acute DSS-induced colitis model [121].

Despite the progress achieved by introducing pH-responsive properties into NPs, several challenges remain, particularly regarding targeting the inflamed intestinal tissues of IBD patients. The gastrointestinal pH profile in patients with IBD is highly dynamic and can significantly influence the performance of pH-sensitive drug delivery systems. Clinical studies have shown that colonic pH values are not constant but fluctuate depending on disease activity, with active inflammation being associated with lower colonic pH compared to remission phases [122]. This acidification is largely attributed to microbial metabolism and local inflammatory processes, which further complicate predictable drug release.

Beyond disease activity, interindividual variability plays an equally important role, as differences in diet, intestinal microbiota composition, and metabolite production contribute to substantial fluctuations in luminal pH [123]. Consequently, pH-sensitive systems must be carefully designed to account for both intra- and interindividual variability, as these factors can determine whether nanoparticles achieve targeted release or undergo premature degradation [124].

pH-sensitive nanoparticles represent a promising approach for targeted drug delivery in IBD; however, their success remains closely tied to the dynamic variations in gastrointestinal pH associated with both disease activity and interindividual differences. These fluctuations highlight the need for further exploration of more adaptable or hybrid designs that can reliably overcome such variability, leaving open the possibility for future innovations to enhance their therapeutic performance.

##### ROS-Responsive NPs

ROS-responsive NPs have gained increasing attention in IBD therapy due to their ability to exploit the elevated oxidative stress characteristic of inflamed intestinal tissues. In the context of IBD, excessive production of ROS such as hydrogen peroxide, superoxide anion, and hydroxyl radicals contributes to mucosal injury and chronic inflammation, creating a pathological microenvironment that can be harnessed for targeted drug release. By incorporating ROS-sensitive linkages, such as thioketal groups, nanocarriers remain stable under physiological conditions but undergo rapid degradation in response to high ROS levels, thereby enabling site-specific delivery and minimizing systemic exposure [125].

Thioketal-based nanoparticles (TKNPs) have been extensively investigated for targeted drug delivery in IBD. Shrestha et al. [126] developed TKNPs (hydrodynamic diameter of ~220 nm and ZP of −21 mV) for delivery of tubastatin A, a potent HDAC6 inhibitor, in models of UC. These NPs remained stable under physiological conditions but underwent rapid degradation in the presence of high ROS levels at inflamed colonic sites, enabling selective drug release. The study demonstrated significant reduction in inflammation and promotion of mucosal healing, highlighting the therapeutic potential of TKNPs in IBD. Xiong et al. [127] investigated ROS-triggered cleavage of thioketal containing supramolecular nanoparticles for oral delivery of dexamethasone. The nanoparticles released the drug selectively at the inflamed colon, effectively alleviating symptoms in both acute and chronic colitis models. TKNPs can be used to conjugate drugs to nanoparticle surfaces, form prodrugs, or control the opening of drug-release gates, overcoming limitations of conventional drug delivery systems, such as uncontrolled release and off-target effects, making them highly suitable for inflammation-targeted therapies [128].

Mesoporous silica as a basis for ROS-responsive NPs continues to evolve, especially via mesoporous organosilica NPs (MON) that degrade under oxidative conditions while also performing other functions in IBD therapy. In one strategy, MONs bridged with diselenide bonds were functionalized with polyethylenimine (PEI) to produce MON-PEI, which exhibits dual scavenging activity binding of pro-inflammatory cell-free DNA (cfDNA) and ROS-responsive matrix degradation. When administered orally in mouse models of ulcerative colitis and Crohn’s disease, MON-PEI preferentially accumulated in the inflamed colon, reduced cfDNA- and ROS-mediated inflammation, ameliorated body weight loss and histologic damage, and lowered inflammatory cytokines, demonstrating efficacy even in delayed treatment settings [129]. Another example involves biosilicification-templated MONs incorporating PEI and L/D-tartaric acid (L/D-TA) complexes (PEI-L/D-TA@MON). These NPs are designed to address multiple triggers in the IBD inflammatory cascade: silanol groups adsorb lipopolysaccharides (LPS), tetrasulfur bonds are redox-sensitive to ROS, PEI binds cfDNA, and the chiral L/D-TA domains provide conformational matching to diseased mucosa to enhance targeting. After oral administration, these particles effectively reduced LPS, ROS, and cfDNA levels, had anti-inflammatory effects, and helped maintain immune homeostasis and microbiota balance [130].

##### Enzyme-Responsive NPs

Enzyme-responsive NPs (ESNPs) have emerged as a promising strategy in IBD therapy, taking advantage of the overexpression of specific enzymes in inflamed intestinal tissue and dysregulated microbiota. This targeted approach has been exemplified by HA-modified NPs co-loaded with curcumin and cerium oxide. When exposed to simulated gastric, intestinal, and colonic fluids, these NPs exhibited significant hyaluronidase-mediated degradation, predominantly in the colonic fluid, thereby demonstrating their responsiveness to colonic enzymes [131]. Beyond conventional small-molecule delivery, ESNPs have been adapted to carry biologically active enzymes or catalytic nanozymes that directly modulate inflammatory pathways. Polymeric NPs functionalized with DNase-I, for instance, have been shown to degrade neutrophil extracellular traps (NETs), thereby reducing neutrophil infiltration, attenuating colonic inflammation, and maintaining integrity of the colon in dextran sulfate sodium-induced colitis [132].

Collectively, these studies illustrate the versatility of enzymatic responsiveness, which can be tailored either for enhanced pharmacokinetics of established drugs or for the delivery of emerging biological therapeutics.

#### 5.1.4. Ligand-Receptor-Mediated Targeting

Inflamed intestinal mucosa provides a unique microenvironment where multiple receptors and adhesion molecules, such as CD44, integrins, MUC5AC, and cytokine receptors, are upregulated and can be exploited to selectively deliver therapeutic agents (Table 3) [106]. For example, chondroitin sulfate-modified nanocarriers designed to bind CD44 have demonstrated preferential accumulation in inflamed lesions and improved disease modulation in preclinical colitis models [133].

Another important axis is the TL1A–DR3 pathway, a ligand–receptor interaction increasingly recognized as a central mediator of chronic intestinal inflammation [34]. Therapeutic strategies that block TL1A or interfere with the TL1A–DR3 signaling cascade have therefore gained momentum. Clinical and preclinical studies suggest that TL1A antagonism not only dampens mucosal inflammation but may also exert anti-fibrotic effects, addressing one of the major complications in CD [134]. These findings position the TL1A–DR3 pathway as one of the most promising ligand–receptor systems for therapeutic intervention in IBD.

Recent reviews further emphasize the potential of less conventional receptors such as PepT1, CD98, and dectin-1, whose upregulation during intestinal inflammation offers additional opportunities for oral, ligand-directed therapies [135].

**Table 3:** Overview of receptor-targeted nanoparticles for inflammatory bowel disease.

Target Cell/Tissue	Receptor	Ligand	Nanoparticle Type	Cargo	Disease/Model	References
Colonic epithelial cells and macrophages	PepT1	KPV	Self-assembly FK506/KPV NPs	Tacolimus (FK506)	DSS-induced colitis	[136]
Activated macrophage	CD44	Hyaluronic Acid (HA)	Self-assembly TWD NPs	Astaxanthin (ASX)	DSS-induced colitis	[137]
Intestinal epithelial cells and macrophages	IL-6R	N/A	Self-assembly BBR/PLGA NPs	Berberine (BBR)	DSS-induced colitis	[138]
Intestinal epithelial cells and macrophages	Somatostatin (SST) and mannooligosaccharide (MOS)	SST and MOS	Self-assembly EUP-Se/SST and MOS NPs	*Eucommia ulmoids* polysaccharides	DSS-induced colitis	[139]
Monocyte-derived macrophages and neutrophils	Integrin	RGD, cRGD, and functional groups	PEG-Lipid-PLGA-based HNPs/NH2/-COOH/RGD/cRGD	BRP-201	In vitro: human monocyte-derived macrophages and neutrophils	[140]
Colonic and intestinal epithelial cells	Manose receptors (CD206)	Manose (M)	M-Se NPs	Selenium (Se)	DSS-induced colitis	[141]
Intestinal epithelial cells and macrophages	CD44	HA	Self-assembly hydrogel-HA-PLGA bilirubin NPs	Bilirubin	DSS-induced colitis	[142]
Macrophages	CD44	HA	IO, ZIF-8/HA NPs	Iron Oxide (IO)	DSS-induced colitis	[143]
Inflamed gut	JAK	N/A	TFC PLGA NPs	Tofacitinib (TFC)	DSS-induced colitis	[144]

Abbreviations: ASX: astaxanthin; BBR: berberine; CD206: cluster of differentiation 206; CD44: cluster of differentiation 44; cRGD: cyclic arginyl-glycyl-aspartic acid peptide; DSS: dextran sodium sulfate; EUP: *Eucommia ulmoides* polysaccharides; FK506: tacrolimus; HA: hyaluronic acid; HNPs: hybrid NPs; IL6R: interleukin-6 receptor; IO, ZIF-8/HA NPs: iron oxide-incorporated zeolitic imidazolate framework-8 hyaluronic acid nanoparticles; M: manose; MOS: mannooligosaccharides; N/A: not applicable; NPs: nanoparticles; PepT1: peptide transporter 1; Se: selenium; SST: somatostatin; TFC: Tofacitinib; TWD NPs: mitochondria-targeted nanoparticles.

#### 5.1.5. Multi-Targeting NPs

Cell-specific targeting strategies have gained increasing attention in IBD therapy, aiming to overcome the heterogeneity of the intestinal microenvironment and to deliver drugs directly to pathogenic cell populations.

Among the most explored approaches, macrophage-targeted systems have shown promise. For example, mannosylated inulin-based NPs demonstrated selective uptake by activated macrophages through the mannose receptor, a surface molecule that is overexpressed on pro-inflammatory macrophages during intestinal inflammation. This receptor-mediated recognition significantly enhanced the internalization of the NPs compared to non-mannosylated controls, ensuring that the therapeutic payload was preferentially delivered to the immune cells most responsible for driving mucosal injury. Moreover, by sparing non-target cells, this approach minimized potential off-target effects and systemic toxicity, highlighting the therapeutic advantage of macrophage-selective nanocarriers for IBD therapy [145]. Building on this concept, Mow et al. [146] introduced a novel lipid nanoparticle formulation designed for oral administration that was capable of targeting both colonic epithelial cells and macrophages. In healthy tissue, epithelial uptake predominated, supporting mucosal delivery and barrier restoration, while in inflammatory conditions macrophage uptake was significantly increased, thereby addressing immune-driven inflammation. This dual-targeting strategy effectively exploited disease-dependent micro-environmental changes, achieving localized drug accumulation, attenuating colonic damage, and maintaining an excellent safety profile.

Beyond macrophages, epithelial cells of the colon also represent an attractive target for drug delivery. A recent study developed a PepT1-targeted nanodrug based on the co-assembly of an anti-inflammatory peptide and an immunosuppressant, which selectively interacted with the PepT1 transporter on epithelial cells. This system effectively restored barrier integrity by enhancing the expression of tight junction proteins such as Claudin-5, Occludin-1, and ZO-1, while simultaneously attenuating colitis symptoms in murine models [136]. Similarly, microRNA-200-loaded lipid NPs have been shown to promote epithelial regeneration by restoring intestinal stem cell function in damaged mucosa, highlighting their potential in therapies aiming to rebuild the epithelial compartment [147].

Recent studies have provided insights into how modulating goblet cell activity can ameliorate disease. For example, autophagy activation (Beclin-1-mediated) in mice reduces endoplasmic reticulum (ER) stress in goblet cells, resulting in thicker, more protective mucus layers and attenuated inflammation. Specifically, constitutive activation of autophagy via Beclin-1 alleviates ER stress in goblet cells, leading to enhanced mucus secretion and a more robust intestinal barrier. This process is microbiota-dependent and requires the activity of the intracellular sensor NOD2. Furthermore, overproduction of mucus alters the gut microbiome, expanding mucus-utilizing bacteria such as *Akkermansia muciniphila*, and protects against chemical- and infection-driven intestinal inflammation [148].

Targeting strategies that exploit endothelial activation have shown promise. For example, cyclosporine A NPs coated with cell membranes carrying integrins α4β1 or α4β7 exhibited higher affinity and uptake by endothelial cells, favoring retention in inflamed intestinal sites and achieving superior therapeutic effects compared to uncoated nanoparticles [149].

## 6. Toxicity and Safety Concerns of IBD-Selective-Nanoparticulate Systems

Despite the significant progress achieved in the nanomedicine field in recent years, toxicity and safety concerns remain hurdles to its clinical translation. Little is known about the toxicity of NPs on the GIT tract. The potential toxic effects of orally administered NPs may be exerted directly on intestinal mucosa or on other tissues and organs of the body following systemic absorption [150]. The local toxicity of NPs can affect the mucus barrier, gut microbiota, epithelial cells, tight junctions, and immune cells [151,152]. Conversely, physiological or pathological factors can induce changes in NPs, rendering toxicity or loss of biological activity [153]. This is particularly important when evaluating the toxicity and safety of nanosystems targeting inflamed intestinal tissues, since the disease is already present and potential toxicity could worsen the inflammation. Furthermore, the modifications made to obtain targeted NPs including size, surface charge, and surface modifications (e.g., PEGylation), as well as the materials used to obtain targeted features (e.g., pH- or ROS-responsive polymers), potentially lead to changes in their pharmacokinetic and toxicological profile [154,155]. In vitro cell culture models are commonly used to assess the toxicity of IBD-targeted NPs against intestinal (e.g., Caco-2) and immune cells (e.g., THP-1) [115,116]. More complex 3D intestinal inflamed models better mimicking an in vivo-like intestinal inflammation environment may be useful used to assess molecular mechanisms of NP toxicity [156]. Limited in vivo biodistribution and safety studies in preclinical animal models, particularly in murine models, give some insights regarding the distribution of targeted IBD NPs in the body and their safety profile [94,96]. The loading of Cyclosporine A (CsA) into mucus-penetrating PEGylated PLGA NPs (CsA-MNP) led to a reduction of one quarter of its systemic exposure after a single oral administration to rats at a toxic dose of CsA ((Neoral^®^, Novartis Pharmaceuticals Corporation), 10 mg-CsA/kg). After daily administration of the same dose of CsA-MNP for 9 days, no significant changes were observed in plasma biomarkers, blood urea nitrogen (BUN), and serum creatinine levels, while these biomarkers were increased in the Neoral^®^-treated group [94]. However, one limitation of the study was the use of healthy animals to assess the potential systemic exposure and kidney damage of CsA-MNP. The presence of disease may influence the pharmacokinetic profile of CsA-MNP. Qassem et al. [96] evaluated the safety profile of lipid nanoparticles (LNPs) composed of newly synthesized ionizable lipids (Lipid 15, ~70 nm, near-neutral surface charge) following retro-orbital injection into a DSS-induced colitis mouse model. Twenty hours later, the levels of the hepatic enzymes alkaline phosphatase (ALP), alanine transaminase (ALT) and aspartate transaminase (AST) in the animals treated with LNA-loaded LNPs and unloaded LNPs remained unchanged and were similar to those of healthy animals.

Translating animal data into human treatments entails significant complexity, and this aspect is even more striking when animal models of disease are used, as in the case of IBD models. The pathophysiological differences between IBD murine models and human IBD, as well as the cross-species differences in biocompatibility and safety, may result in different NP safety profiles when translated into humans [7]. In addition, NPs for IBD treatment warrant long-term safety evaluations. This is particularly important due to the chronic nature of the disease and the need for long-term administration [157].

## 7. Conclusions and Future Perspectives

Nanoparticle-based drug delivery systems (NPs) have demonstrated remarkable potential in the preclinical management IBD. Their unique physicochemical properties, including small size, tunable surface modifications, and capacity to encapsulate diverse therapeutic cargos, enable site-specific delivery, controlled release, and reduced systemic toxicity compared to conventional formulations. Recent studies have highlighted their ability to accumulate at inflamed intestinal sites by exploiting pathophysiological features such as pH shifts, enzymatic activity, and oxidative stress [84].

Future research directions point toward multifunctional and precision-driven approaches. The development of multi-targeting NPs capable of acting simultaneously on immune and epithelial compartments could yield more comprehensive therapeutic effects [158].

Despite significant progress, no nanomedicine-based IBD drug delivery system has entered clinical trials to date [159]. Several hurdles remain to be overcome before intestinal inflammation-targeted nanosystems can be translated into clinical practice. Some of the challenges to be addressed in the next chapters of nanomedicine’s clinical translation include regulatory constraints, scalability issues, protocol standardization, and the need for long-term safety data in humans [160,161].

In conclusion, nanomedicine represents a paradigm shift in the treatment of IBD. By addressing the current limitations of conventional therapies, NPs hold the potential to improve therapeutic efficacy, minimize adverse events, and enable personalized interventions. Continued interdisciplinary collaboration among material scientists, gastroenterologists, immunologists, and regulatory authorities will be essential to bridge the gap between preclinical promise and clinical application. With sustained innovation and rigorous translational research, nanoparticle-based systems are expected to become a cornerstone in the future management of IBD.

## Data Availability

No datasets were generated or analyzed during the current study.

## Abbreviations

CAM	Cell Adhesion Molecules
CD	Crohn’s Disease
cfDNA	Cell-free DNA
DRA	Downregulated in Adenoma
DR3	Death Receptor 3
ECCO	European Crohn’s and Colitis Organization
eEPR	Enhanced Epithelial Permeability and Retention
ER	Endoplasmic Reticulum
ESNPs	Enzyme-responsive Nanoparticles
GI	Gastrointestinal tract
IBD	Inflammatory Bowel Disease
IECs	Intestinal Epithelial Cells
IL	Interleukin
IV	Intravenous
JAK	Janus Kinase
L/D-TA-L/D	Tartaric Acid
LNPs	Lipid-based Nanoparticles
LPS	Lipopolysaccharides
MON	Mesoporous Organosilica Nanoparticles
MMPs	Matrix Metalloproteinases
MNPs	Metallic Nanoparticles
NETs	Neutrophil Extracellular Traps
NHEs	Sodium Hydrogen Exchangers
NLCs	Nano Structured Lipid Carriers
NOD2	Nucleotide-binding Oligomerization Domain-containing protein 2
NPs	Nanoparticles systems
PEI	Polyethylenimine
PLNs	Polymer–Lipid Hybrid Nanoparticles
ROS	Reactive Oxygen Species
SC	Subcutaneous
SCFAs	Shory-Chain Fatty Acids
SLNs	Solid Lipid Nanoparticles
S1P	Sphingosine 1-phosphate
TfR	Transferrin Receptors
TGF-β	Transforming Growth Factor β
Th1	Type 1 helper-T-cell
Th2	Type 2 helper-T-cell
TKNPs	Thioketal-based Nanoparticles
TL1-A	TNF Ligand-related molecule 1A
UC	Ulcerative Colitis
